# Prospects for Anti-Biofilm Pharmaceuticals

**DOI:** 10.3390/ph8030504

**Published:** 2015-08-27

**Authors:** Philip S. Stewart

**Affiliations:** Center for Biofilm Engineering and Department of Chemical and Biological Engineering, 366 EPS Building, Montana State University, Bozeman, MT 59717, USA; E-Mail: phil_s@biofilm.montana.edu; Tel.: +1-406-994-1960; Fax: +1-406-994-6098

**Keywords:** biofilm, drug, antibiotic

## Abstract

This commentary highlights several avenues currently being pursued in research labs to the development of new anti-biofilm pharmaceuticals. There is a real need for alternative therapeutic modalities for treating the persistent infections that sometimes form on implanted medical devices or compromised niches within the body. Strategies being researched include discovering new antimicrobial agents that kill microorganisms in biofilms more effectively than do existing antibiotics, designing drugs that block microbial adhesion or interfere with intercellular communication, developing chemistries to disperse biofilms, and combining agents with different mechanisms of action. Though the need is great, the pathway to commercialization of new drugs is steep. One possible streamlined approach to navigating the regulatory approval process is to repurpose old drugs, a strategy that a few groups have shown can yield agents with anti-biofilm properties.

## 1. Introduction

Microbial biofilms are at the root of diverse persistent infections such as those associated with prosthetic joints, cystic fibrosis pneumonia, urinary and central venous catheters, periodontitis, and chronic dermal wounds [1]. These infections tend to be localized, slow-moving, recalcitrant to antimicrobial chemotherapy, and recurrent [2]. They take a huge toll on patients, physicians, and health care staff and they pose a large economic burden. Can new pharmaceuticals help mitigate this problem? One reason to answer “yes” to this question is that microorganisms in biofilms present many novel drug targets because the activities and pathways of microorganisms in a biofilm are distinct from those that are important in free-floating microbial cells [3]. This article examines some of the unique targets that biofilms afford and illustrates the potential for drug discovery with a few examples [4,5,6]. It was prepared in a short format that was intended to be thought provoking rather than comprehensive.

## 2. New Drugs that Kill Microorganisms in Biofilms

Microorganisms in biofilms are commonly found to be highly tolerant of conventional antibiotic treatment [7]. The antiseptics and antibiotics available to health care practitioners have all been discovered, optimized, and commercialized using planktonic methods to assay antimicrobial activities. It is not surprising that agents developed for planktonic targets perform suboptimally against biofilm targets. One of the first suggestions that can be made is simply to use biofilm-based methods in the search for new drugs. There are many well-characterized biofilm methods these days including screening methods such as microtiter dish assays [8] and continuous flow reactors such as the drip-flow biofilm reactor [9] and CDC biofilm reactor [10].

Most extant antibiotics target some macromolecular biosynthetic process such as cell wall synthesis, DNA replication, or translation. In a cell where these processes are inactive, inhibiting the process may not induce lethal cell damage. Biofilms are known to harbor physiological heterogeneity; they can contain both actively growing cells and, a short distance away, cells that have entered a non-growing or dormant state [11,12,13,14]. These non-growing cells are often referred to as persisters for their ability to survive stresses that eliminate growing cells, though there may not be an exact correspondence between the non-growing and persister states. One strategy for defeating a biofilm infection is to find agents that more effectively target non-growing or persister cells [15]. For example, Podos *et al.* [16] described a novel antimicrobial agent that kills staphylococci in stationary phase more effectively than conventional antibiotics. An acyldepsipeptide derivative has been reported to kill persister cells by activating an endogenous protease: then persisters self-digest [17]. A third example is an anticancer drug, mitomycin C, that irreversibly crosslinks DNA in a process that does not depend on active growth of the cell [18]. Finally, it should be possible to potentiate the action of a conventional antibiotic by providing appropriate metabolic stimuli to “wake-up” persister cells [19].

Another class of antimicrobial agents with potential to target dormant cells is antimicrobial peptides and peptide-mimics [20,21]. These agents work by permeabilizing the cell membrane. An intuitively appealing approach to preventing biofilm infection of an implanted medical device is to coat or bind antimicrobial peptides to biomaterial surfaces [22,23]. Some peptides appear to have multifactorial anti-biofilm activity above and beyond their bactericidal activity [24,25,26,27]. For example, peptides have been reported to promote detachment [24], or interfere with attachment [25], quorum sensing [25], or the bacterial stress response known as the stringent response [27].

## 3. New Drugs that Block Microbial Adhesion

The first step in the formation of a biofilm is attachment or adhesion of a cell to a surface, either epithelial or biomaterial. Blocking this initial step could be an effective means of preventing biofilm infection. A sophisticated pharmaceutical version of this approach is emerging in the context of urinary tract infections caused by enteric bacteria. These microorganisms rely on proteinaceous appendages such as pili and curli to adhere. As detailed molecular pathways of pilus and curli biosynthesis, export, and assembly have been elucidated it has become possible to identify inhibitors of these processes. These agents, sometimes termed pilicides or curlicides, target the bacterium and the biofilm very specifically [28] and have demonstrated potential in animal models [29].

## 4. New Drugs that Interfere with Microbial Communication

One of the most heralded alternative therapeutic approaches for targeting biofilms has been disruption of intercellular communication. Immediately after the seminal paper linking quorum sensing and biofilm formation appeared in 1998 [30], it was broadly conjectured that jamming bacterial communication would stop a biofilm in its developmental tracks. The prospect of quorum sensing inhibitors has enjoyed sustained enthusiasm in the popular and technical press ever since [31]. Examples of signaling molecules for which a biofilm connection has been investigated include homoserine lactones [32], cis-2-unsaturated fatty acids [33], and a furanosyl borate diester [32]. The normal functioning of these systems can be disrupted by either agonists or antagonists of the native signal molecule. There are indeed many reports of small molecules that interfere with quorum sensing and could be drug candidates [34,35,36]. From the kitchen cupboard alone, compounds or extracts from guava, garlic, horseradish, coffee, cloves, vanilla, and ginger have been reported to disrupt homoserine lactone-based signaling. Many synthetic antagonists have been described as well. It is not a lack of candidate molecules that limits the conversion of quorum sensing inhibitors into pharmaceuticals. Some of the possible challenges to commercializing these chemistries are touched on below in Section 7.

## 5. New Drugs that Disperse Biofilms

Because microorganisms in a biofilm can be so protected from killing by antimicrobial agents, an attractive alternative strategy is to take aim at the biofilm matrix. Breaking the biofilm apart could increase the susceptibility of released cells, and possibly also the residual biofilm cells, to host defenses or conventional antiseptics and antibiotics. The biofilm matrix is thought to be composed primarily of extracellular polysaccharides, proteins, and nucleic acids [37]. These extracellular polymeric substances (EPS) could be targeted directly, or the cellular processes required for their synthesis could be interdicted.

A very straightforward approach to dispersing a biofilm is to deploy enzymes that degrade the polymers that make up the biofilm matrix. Bacteria released from a biofilm typically become more susceptible to antimicrobial attack and so might be controlled by conventional antibiotics or the host defenses. Polysaccharide lyases [38] and DNAses [39,40] are two examples of enzymes that have been shown to break down EPS in certain biofilms.

A wide variety of chemistries have been reported to have biofilm-dispersal activity [41,42,43,44,45]. One particularly intriguing target is regulation mediated by the secondary messenger molecule cyclic di-GMP. This molecule appears to mediate a switch between a sessile or biofilm mode of growth and a motile, planktonic mode of growth [46,47]. Many bacteria share this regulatory mechanism and in all organisms studied so far it operates in a similar manner. High levels of cyclic di-GMP lead to synthesis of EPS and promote biofilm formation whereas low levels of cyclic di-GMP lead to breakdown of the matrix and cell release. Thus, agents that interfere with cyclic di-GMP metabolism or its binding to target proteins could induce biofilm dispersal. Medicinal chemists have begun to identify such compounds [48,49]. Other chemical signals tied in with cyclic di-GMP, such as nitric oxide, might also induce dispersion [50].

## 6. Combination Therapies

A recurrent theme in the discussion of anti-biofilm technologies is the likely need for combination approaches. Because biofilms are so resilient, heterogeneous, and often polymicrobial, it may be necessary to combine technologies that work by different mechanisms of action. For example, quorum sensing antagonists or agonists by themselves probably will not eliminate a biofilm. If they render the biofilm more susceptible to antimicrobial chemotherapy, such combinations could be useful [51]. A similar argument applies to dispersing agents, for which combination with an antibiotic may be important not only to eliminate the residual attached cells but to control the cells released from the biofilm. Even bacteria of the same species may require a two-pronged attack if subpopulations of cells enter distinct physiological states. For example, the persister-killing protease activator mentioned previously [17] shows synergy with a conventional antibiotic, rifampicin, in a mouse model of infection. The combination of agents limits the accumulation of resistant mutants and it is likely that one agent is more effective against dormant cells while the other is more effective against growing cells.

## 7. Translational Pathways

It should be clear from the preceding survey that there are abundant ideas, and quite a few candidate molecules, for developing new anti-biofilm pharmaceuticals. We are not idea limited. The hard part is translating these ideas into commercial reality. To illuminate the challenges, consider the rocky road that inventors of quorum sensing inhibitors (see Section 4) have travelled. A half-dozen small companies were started, by bright scientists, to bring quorum sensing inhibition technology to market. None of these companies has succeeded and most have gone under. Why has this path been so treacherous? The very short answer is that there are complex scientific, technical, cultural, regulatory, diagnostic, and intellectual property hurdles to commercializing biofilm-targeting pharmaceuticals. The lesson that can be drawn is that it is difficult to commercialize a new drug and this reality should be soberly and realistically engaged by those attempting to pursue any of the innovative strategies discussed above.

The investment of time and money required to obtain regulatory approval for a new drug is daunting. Repurposing old drugs may offer a clever short-cut. This can be done by screening libraries of existing pharmaceuticals, obviously originally approved for uses other than treating biofilms, for their anti-biofilm activity. Several groups have reported success with this approach [49,52,53,54] and the deployment of the anticancer drug mitomycin C [18] against biofilms would be another example.

Awareness of the biofilm infection paradigm is growing in the medical community at the same time researchers are describing an expanding portfolio of anti-biofilm technologies. These convergent trends foretell a day, not too distant, when new pharmaceuticals will enhance treatment options for the recalcitrant, debilitating infections that stem from microbial biofilms.
